# Gym and Fitness Injuries amongst those Aged 16–64 in New Zealand: Analysis of Ten Years of Accident Compensation Corporation Injury Claim Data

**DOI:** 10.1186/s40798-024-00694-9

**Published:** 2024-05-14

**Authors:** Melissa Cuthbertson-Moon, Patria A. Hume, Hannah E. Wyatt, Isaac Carlson, Bryce Hastings

**Affiliations:** 1 Sports Performance Research Institute New Zealand (SPRINZ), Auckland University of Technology, Auckland, New Zealand; 2 Present Address: Auckland Bioengineering Institute, The University of Auckland, Private Bag 92019, Auckland, 1142 New Zealand; 3 Mindaroo Tech & Policy Lab, Law School, The University of Western Australia, Perth, Australia; 4 Accident Compensation Corporation, Wellington, New Zealand; 5 Les Mills International, Chicago, USA; 6 Present Address: Faculty of Health, University of Canterbury, Christchurch, New Zealand

**Keywords:** Injury, Sports, gym, Fitness, Soft tissue injury

## Abstract

**Background:**

To provide epidemiological data for minor and moderate-to-serious injury claims for gym and fitness related injuries amongst those aged 16–64 in New Zealand, to inform the development of an injury prevention program.

**Methods:**

Retrospective analytical review of gym and fitness related injury entitlement minor and moderate- to-serious Accident Compensation Corporation (ACC) claims from 1 July 2011 to 30 June 2020. Data were analysed by cause of injury, geographical region, sex, age, body site and injury type. Qualitative analysis of free text describing the activity causing the injury was conducted.

**Results:**

Over the ten-year period, 16–64 year olds made 345,254 injury claims, costing ACC NZ$241,298,275 in treatment charges. Soft tissue injuries were the most prevalent making up 96% (331,343) of all claims and 88% (NZ$213,049,197) of the total charges. Strenuous movement with lifting (*n* = 154,467, 47%), strenuous movement without lifting (*n* = 84,469, 25%), impact/contact with object (*n* = 39,610, 12%) and impact/contact with ground (*n* = 25,351, 8%) were the top four mechanisms resulting in injury, accounting for 92% of soft tissue injuries. Males and females aged 21 to 30 years old were most frequently injured. The four most injured body sites (lower back/spine, shoulder, knee, neck/back of head) accounted for 63% of injuries in females, and 65% in males.

**Conclusions:**

The most common cause of injury from gym and fitness activity claims in 16–64 year olds in New Zealand was lifting/carrying/strain resulting in lower back/spine and shoulder (including clavicle/blade) soft tissue injuries. Soft tissue injuries accounted for 96% of the total claims. Males and females aged 21 to 30 years old were most frequently injured age group.

**Supplementary Information:**

The online version contains supplementary material available at 10.1186/s40798-024-00694-9.

## Key points

• The most common cause of injury from gym and fitness activities amongst those aged 16–64 was lifting/carrying/strain resulting in lower back/spine and shoulder soft tissue injuries.
• Of all injuries, 96% were soft tissue injuries.
• Males and females aged 21 to 30 years old were most frequently injured.

**Supplementary Information:**

The online version contains supplementary material available at 10.1186/s40798-024-00694-9.

## Background

Physical activity is an important aspect of maintaining a healthy lifestyle and public gyms, fitness clubs and home gyms are a popular location to perform exercise. In New Zealand (NZ), the number of gym and fitness training related injury claims were reported greater than every other sport [1]. In 2020 the Accident Compensation Corporation of New Zealand reported that gym and fitness claims surpassed the number of claims for New Zealand’s national sport of rugby [1]. An Accident Compensation Corporation (ACC) report showed an 18% increase in gym injuries between 2015 and 2020 with the cost of support rising by 43% [2]. New Zealand has a nationwide sports injury prevention program led by ACC named SportSmart which has sport specific adaptions including RugbySmart [3, 4], and SoccerSmart [5, 6]. No such program for recreational gym injuries exists. Given that in 2019 the NZ Register of Exercise Professionals (REP’s) reported that 500,000 people in New Zealand are members of an exercise facility (gym) [7], a gym specific injury prevention programme is warranted due to an expected rise in global [fitness] club memberships, with a predicted compound annual growth rate of 7.21% between 2022 and 2027 [8]. A large amount of resourcing was put into RugbySmart to address the more serious and costly injuries associated with the sport, NZ Rugby reported 160,000 registered rugby players in New Zealand [9]. Whilst gym and fitness injuries are less severe and less costly than those for rugby participation it is desirable that those who participate in any activity do not become injured because of their engagement [10, 11]. Our aim is to use ACC claims data to inform an injury prevention program for gym and fitness injuries.

ACC is responsible for providing injury compensation and rehabilitation services to all New Zealand citizens and visitors who incur an accidental personal injury and make a claim at the time they seek medical treatment, through a registered health provider in New Zealand. The national no-fault injury compensation system managed by ACC means that New Zealand is in a unique position whereby epidemiological data on sports injuries including treatment costs are recorded [12]. ACC’s injury data can provide valuable insights into characteristics and trends of gym-related injuries. Data that ACC gather includes type of injury, location, and cost of treatment, and can be used to inform policies and strategies for injury prevention [13]. Cover includes medical expenses, lost earnings, support for recovery and rehabilitation [14].

The aim of this work was to deep dive the ACC database of gym and fitness related injury claims to determine the most common injuries incurred in a recreational gym environment, to inform the development of an injury prevention strategy. ACC injury data between 1 July 2011 and 30 June 2020 were analysed to examine characteristics and trends of gym and fitness related injuries. We were able to make comparisons of injury type and body site, compile cost charges associated with treatment of the injuries, and perform an analysis of “accident description” free text narrative.

## Methods

### Ethical Consent

ACC claims data are considered confidential and personal information is only used for authorized purposes in accordance with the Privacy Acts of 1993 and 2020 [15, 16] and the Accident Compensation Act 2001 [17]. The research team agreed to ACC’s data confidentiality terms and to refrain from reporting information that could be used to identify any individual, by signing a confidentiality agreement. It was not possible to obtain informed consent from the injured participants as de-identified data were provided to the research team from the ACC database. Ethical consent for the research was obtained from ACC [#CRM:0140013] and the Auckland University of Technology ethics committee [AUTEC #22/22].

### New Zealand ACC Injury Data and Reporting

ACC recorded detailed information at the time of injury reporting using a standard form to ensure consistency in reporting, recording and analysis. ACC reported on two types of acute personal injury claims; minor and moderate-to-serious claims (MSC) [12]. Both were defined under the Injury Prevention, Rehabilitation and Compensation (IPRC) Act, 2001 with ACC responsible for meeting the injury costs. People qualified for cover when they presented with a personal acute injury as a result of an accident to any of the ACC recognised 30,000 registered medical practitioners throughout New Zealand [18]. A claim was classified as ‘minor’ when ACC only paid for medical treatment provided by a registered medical practitioner (e.g., Physiotherapist, General Practitioner). Typically, this involved a few treatments with ACC meeting most costs with claims tending to be strains, sprains, cuts, and bruises. To be classified as moderate-to-serious, these injuries usually required assistance beyond medical treatment alone. Moderate-to-serious claims may involve a combination of medical care, rehabilitation costs and income replacement for employment time lost because of the injury. These types of claims include broken bones, lacerations, head injuries and spinal cord injuries, and they require more extensive treatment and may result in significant time off work or disability. The focus of the claim is to provide medical treatment and rehabilitation to get the injured party back to their usual daily activities as quickly as possible. New injury claims are categorised as those opened within the reporting year, and ongoing injury claims carry on to the next reporting period [12].

For this study, we focused on minor and moderate-to-serious claims that occurred from 1st July 2011 to 30th June 2020 from participation in fitness and gym activities. The definition of injury utilized for this study was “any injury that had been assessed and reported by a registered health practitioner as a result of sports participation” [19]. To be recorded in the study dataset the injury was required to have been classified and recorded within the fitness and gym related injury code and accepted as being an ACC claim during the study period. The ACC database only recorded the activity prior to the accident as ‘recreation/sporting activity’, and not if the claimant was exercising at the time of injury.

We extracted information on the nature of injury, body part injured, equipment involved, and demographic characteristics of the injured gym user. We excluded gym users under the age of 16 who required adult supervision to partake in gym activities [20], and aged 65 years and older. 16 year olds were chosen as the lowest age, because young people aged 16–17 years old are allowed to workout unsupervised and allowed to enter a written gym membership agreement with payment [20]. 61% of young adults aged 16–24 held gym memberships [21]. Persons aged 65 and older were excluded because participation levels have been known to decrease and there is a preference to spend time on other things rather than exercise [22]. We analysed data using descriptive statistics to examine characteristics and trends of gym-related injuries in New Zealand. Total claims were calculated, with sub analyses by sex, age, ethnicity, injury site and type. The total mean and median cost per claim (charge) were calculated for comparison and to aid with data understanding. Supplementary Table 1 provides the data categories obtained from ACC used in analyses.

As there were no reliable participation data collected by the different fitness activities, New Zealand population data were obtained from official government data, which provides estimates of resident populations between each five-year census [23], this information could be used to identify trends within the data. The ACC claims database is a good representation of the injuries sustained in New Zealand. However, the data are not a comprehensive collection of injuries because it is not mandatory to report an injury to ACC. There is no indication of whether injuries reported to ACC were new, old, or recurrent.

### Definitions

The term soft tissue injury is not well defined [24–26], it usually refers to injuries that occur in muscles, tendons and ligaments, however can also refer to injuries of tissue including vascular and nervous tissue [24]. Supplementary Table 2 summarises a selection of descriptions from the literature. The ACC database categorised soft tissue injuries as “soft tissue (not stated)”. For the purpose of discussion we use the American Academy of Orthopaedic Surgeons description of soft tissue injuries as those that are muscular, ligamental, sprains, strains, contusions, tendonitis and bursitis [27].

### Statistical Analyses

All data were entered into a Microsoft Excel spreadsheet and cleaned. Data were analysed with JMP 16 ® for Mac (SAS, released 2021, SAS Institute inc. Cary, NC, USA.), RStudio® for Mac (RStudio, Version 2022.07.1, Posit PBC, Boston, MA, USA), and NVivo® for Mac (NVivo 1.6.2, QSR International Pty Ltd, Burlington, MA, USA.). All costs reported in New Zealand Dollars (NZ$).

Percentages injured by sex and ethnicity were compared with the New Zealand population, based on the 2018 census [23] which reported the New Zealand population of 4.7 million was comprised of NZ European (70.2%), NZ Māori (16.5%), Pacific Peoples (8.1%), Asian (15.1%) and other ethnic groups (2.7%).

The data set contained a field of free text entries of a short description of the accident that was recorded at the time of reporting. Free text of the soft tissue injuries was converted to a word document and analysed with NVivo and JMP. For the 4,286,980 words recorded, a word frequency query (including stemmed words) was performed in NVivo and a word cloud created of the most frequent words.

## Results

Analysis revealed 345,254 gym-related injuries in New Zealand during 1st July 2011 to 30th June 2020 at a cost to ACC of NZ$241,298,275. The MSC injuries recorded for 16–64-year-olds, were 345,254 with approximately equal proportions for females (175,045 claims, 50.7%) and males (170,200 claims, 49.3%). The mean cost per claim was NZ$698, the median cost per claim was NZ$198, and cost range was NZ$5 to NZ$2,647,697.

### Ethnicity

NZ European recorded 221,537 claims accounting for 64.2% of injuries, costing NZ$162,394,179 (67%). NZ Māori recorded 36,560 claims at 10.6% of all reported injuries, with a total claims cost of NZ$24,538,273 (10.2%). Pacific Peoples reported 19,724 (5.7%) injuries costing NZ$12,404,456 (5.1%) and Asian reported 38,569 (11.2%) injuries at a cost of NZ$23,370,042 (9.7%). Other/unknown ethnicity whilst accounting for 2.7% of the NZ population, accounted for 8.4% of the claims (28,864), NZ$18,591,323 in rehabilitation costs with this difference likely owing to ACC covering visitors and residents of New Zealand (Table 1).

**Table 1:** Number of claims per ethnicity

Ethnic group	Population (2018 census)	Population distribution % [43]	Number of claims ^b^	% of claims	% scaled to population ^a^	Cost NZ$	%
NZ European	3,297,864	70.2	221,537	64.1	72.2	162,394,179	67.3
NZ Māori	775,836	16.5	36,560	10.6	11.9	24,538,273	10.2
Pacific Peoples	381,642	8.1	19,724	5.7	6.4	12,404,456	5.1
Asian	707,598	15.1	38,569	11.2	12.6	23,370,042	9.7
Other/ unknown (including MELAA) ^c^	128,385	2.7	28,864	8.4	9.5	18,591,323	7.7
Total	4,699,755	112.6 ^a^	345,254	100	112.6	241,298,275	100

^*a*^ *Percentage totals more than 100 as respondents can select more than one ethnicity*

^*b*^*ACC claims datasets are ethnicity prioritised, where more than one ethnic group is recorded, only a single ethnicity is used in reporting and follows the Statistics NZ prioritisation algorithm* [44]

^*c*^ *ACC data recorded as other/unknown nationality includes MELAA which is recorded as an independent ethnic group in the NZ census*

### Geographical Region

Of the recorded claims 67% were in the three most populated regions, Auckland, Wellington, and Canterbury. Auckland was the region with the highest number of claims 156,035 (45%), Wellington reported the second highest number 40,578 (12%), Canterbury had the third highest number 36,106 (10%) (Supplementary Table 3). Whilst it was expected that most injury claims would occur within the three largest regions for population, the number of claims made by those living in Auckland (45%) were disproportionately higher than the population distribution (33%). Reported sport participation and the number of gyms in the Auckland region are not disproportionately higher than those in Wellington and Canterbury [28, 29], suggesting those living in Auckland may be more likely to make claims than those living in other regions of New Zealand.

### Age and Sex Distribution

Claims were selected for 16–64 year olds in New Zealand, Males were responsible for the largest total cost of claims NZ$126,470,148 (52%) for a lower number (*n* = 170,200, 49%) of claims. Females claimed NZ$ 114,828,127 (48%) for the 175,054 (51%) claims. The most injured age groups were 26–30 years old (53,096 claims; 15%) and 21–25 years old (52,240 claims; 15%) (Table 2).

**Table 2:** Total number of claims and cost by age range and sex (costs in NZ$)

Age range (years)	Total number	Female	Male	Total Cost NZ$	Female NZ$	Male NZ$
16–20	33,705	13,992	19,713	13,836,736	5,811,745	8,024,991
21–25	52,240	23,990	28,250	29,121,659	13,106,508	16,015,151
26–30	53,096	25,060	28,036	33,826,222	15,551,875	18,274,347
31–35	43,625	21,155	22,470	30,018,849	13,607,707	16,411,142
36–40	40,336	20,875	19,461	30,463,799	14,604,724	15,859,076
41–45	37,903	21,128	16,775	30,862,365	15,729,844	15,132,520
46–50	33,316	19,355	13,961	29,800,564	14,732,541	15,068,023
51–55	25,947	15,209	10,738	22,443,562	11,437,189	11,006,372
56–60	16,783	9,688	7,095	14,045,470	7,176,112	6,869,359
61–64	8,303	4,602	3,701	9,299,483	4,394,874	4,904,610
Total	345,254	175,054	170,200	241,298,275	114,828,127	126,470,148

**Fig. 1:**
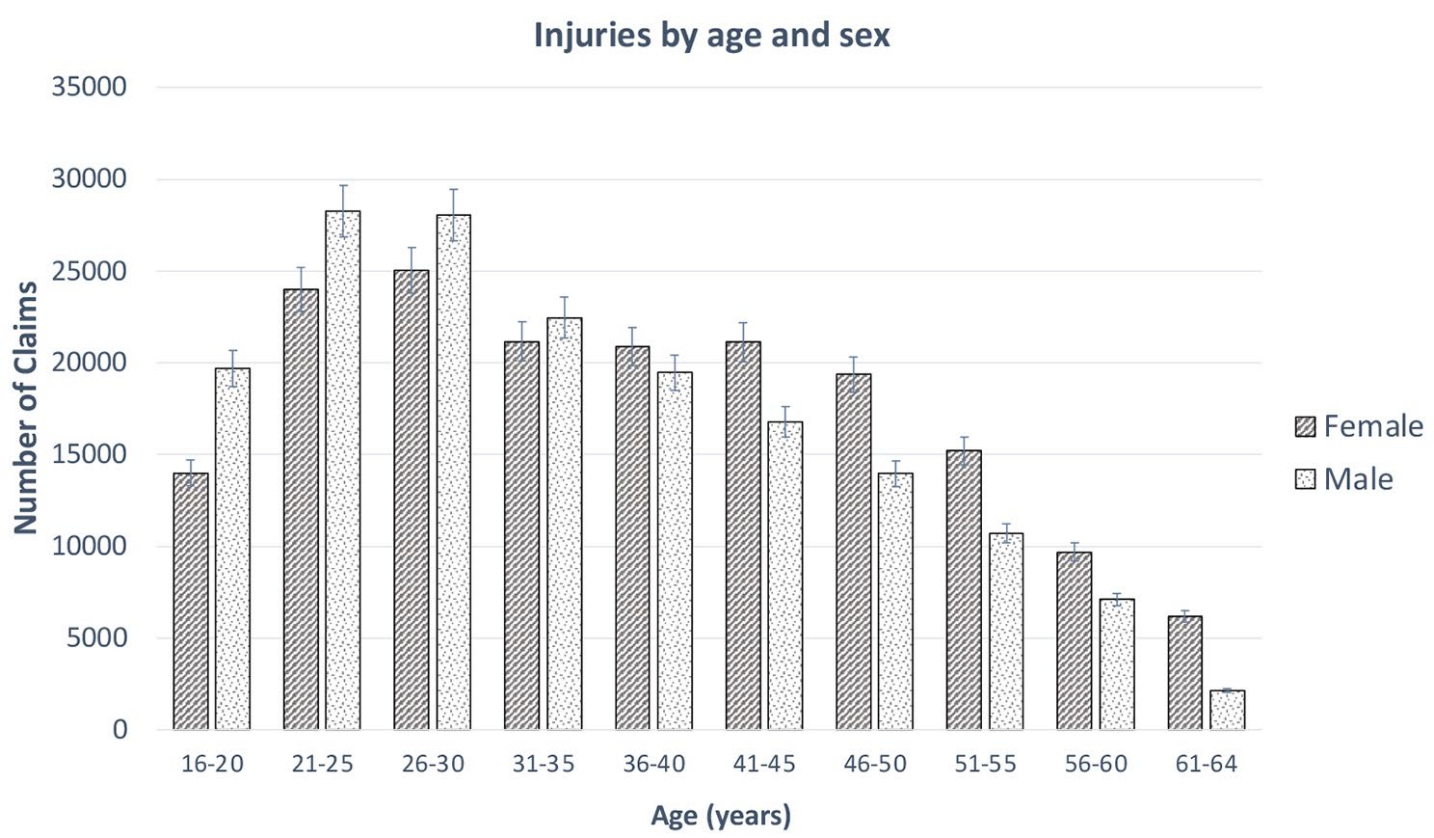
Total number of injury claims for age range and sex (95% confidence interval)

**Fig. 2:**
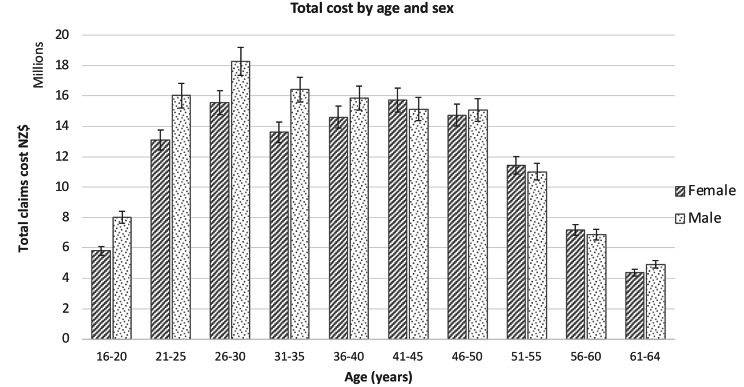
Total cost of injury claims for age range and sex (95% confidence interval)

Analysis by age range and sex for claim numbers (Fig. 1) and cost (Fig. 2) showed that the 26–30 years old age group had the largest total cost of claims (NZ$33,826,222) and largest number of claims for females (25,060). The 21–25 years old age group had the largest number of claims for males (28,250). The 41–45 years old age range had the largest total cost (NZ$ 15,729,844) for females and 26–30 years old age range for males was the largest total cost (NZ$ 18,274,347). Males recorded the largest number of claims (54%) between the ages of 16 and 35 and females recorded the largest number of claims (57%) from the age of 36 years and above.

### Body Parts and Injury Types

Of all claims 96% (*n* = 331,343) were soft tissue injuries (Table 3) predominately caused by strenuous movement with lifting. Fractures and dislocations (*n* = 4088 claims, 1%) and laceration/puncture wounds (*n* = 3424, 1%) accounted for the top three types of injuries reported. Soft tissue injuries accounted for 88% (NZ$213,049,197) of the total costs.

**Table 3:** Frequency of injury type for the n that reported a cause of injury

Injury type	n (from N total = 345,254)	% of claims with cause of injury reported
Soft tissue	331,343	96
Blank (miscellaneous)	6,064	2
Fracture / dislocation	4,088	1
Laceration / puncture wound	3,424	1

Of the soft tissue injuries, the four most injured sites for both males and females accounted for 217,581 claims (66%) and were the lower back/spine (81,799 claims, 24%), shoulder including clavicle/shoulder blade (61,784 claims, 19%), neck/back of head/vertebrae (37,845 claims, 11%) and knee (36,153 claims, 11%) (Supplementary Table 4). The four most injured sites accounted for around 76% of costs (27% lower back/spine NZ$57,111,225; 25% shoulder NZ$52,245,523; 15% knee NZ$31,010,754; 9% neck/back of head/vertebrae NZ$19,125,569). The four most injured sites accounted for 63% of injuries in females, and 65% in males and consisted of the Lower back/spine, Shoulder/clavicle, Knee and Neck/back of head/vertebrae (Supplementary Table 5). The differences in the number of claims between males and females for shoulder/clavicle (males 21%, females 15%) and knee (males 9%, females 13%) is noted. We have no participation data for New Zealand to call upon, however there is still a general trend toward more males participating in strength training activities, likely accounting for the higher number of shoulder injuries. More females in general tend to perform exercise via cardio equipment (treadmill, elliptical trainer, exercise bike) which is likely to account for more knee injuries observed [30, 31].

### Mechanism of Injury

Of the 330,816 soft tissue injury claims recorded, 298,064 (90%) were within four cause categories. Lifting/carrying/strain accounted for 162,598 (49%) of soft tissue claims, loss of balance/personal control was 57,038 (17%), twisting movement was 44,015 (13%) and pushed or pulled was 34,413 (10%) of claims (Table 4). Other or unclear causes (*n* = 9,905, 3%), slipping, skidding on foot (*n* = 8030, 2%), tripping or stumbling (*n* = 3,260, 1%), misjudgement of support (*n* = 2,944, 1%) and collision/knocked over by object (*n* = 2,227, 1%) made up 8% of the total injury claims. Additional mechanisms made up only 2% of the total causes.

**Table 4:** Mechanism of soft tissue injury by cause. Total soft tissue injury claims *n* = 331,343

Cause	n	%
Lifting/Carrying/Strain	162,598	49
Loss Balance/Personal Control	57,038	17
Twisting Movement	44,015	13
Pushed or pulled	34,413	10
Other or Unclear Cause	9,905	3
Slipping, Skidding on Foot	8,030	2
Tripping or stumbling	3,260	1
Misjudgement of Support	2,944	1
Collision/Knocked Over by Object	2,227	1
Weak Property or Characteristics	1,543	0
Struck by Person	1,083	0
Object Coming Loose/Shifting	966	0
Loss of Hold	698	0
Puncture	683	0
Swerving/Evasive Action	559	0
Struck by Held Tool/Implement	234	0
Loss of Consciousness/Sleep	122	0
Folding/Collapse	116	0
Something Giving way Underfoot	104	0
Collapse/Overturning/Inundate	73	0
Collapse of Stack/Bulk Goods	42	0
Explosion/Blasting/Implosion	32	0
Inadvertent Machine/Vehicle Movement	28	0
Mechanical Malfunction	26	0
Skid	26	0
Bursting/Breakage/Distortion	20	0
Recoil/Ejection	19	0
Electrical Shock/Short Circuit	12	0

Strenuous movement with lifting (*n* = 154,467, 47%), strenuous movement without lifting (84,469, 25%), impact/contact with object (*n* = 39,610, 12%) and impact/contact with ground (*n* = 25,351, 8%) were the top four contacts reported, accounting for 92% of the soft tissue injuries (Table 5).

**Table 5:** Mechanism of soft tissue injury by contact. Total soft tissue injury claims *n* = 331,343

Contact	n	%
Strenuous Movement with Lifting	154,467	47
Strenuous Movement without Lifting	84,469	25
Impact/Contact with Object	39,610	12
Impact/Contact with Ground/Floor	25,351	8
Other or Unclear Contact	11,363	3
Repetitive Movement	4,351	1
Caused Own Injury Without Tool	4,172	1
Contact While Handling/Carrying	2,409	1
Other Contact with Person	1,866	1
Dropped Object Carried/Handled	1,000	0
Contact With Object Carried/Handled	453	0
Step on Sharp Object	276	0
Falling Objects Not Handled	133	0
Other Moving Object/Part Etc	265	0
Contact with Moving Object	278	0
Collision	156	0
Blank	175	0
Exposed to Flame/Noise/Elect	14	0
Witnessed	22	0
Environmental Elements	17	0
Flying Object/Spatter/Fragments	5	0

**Fig. 3:**
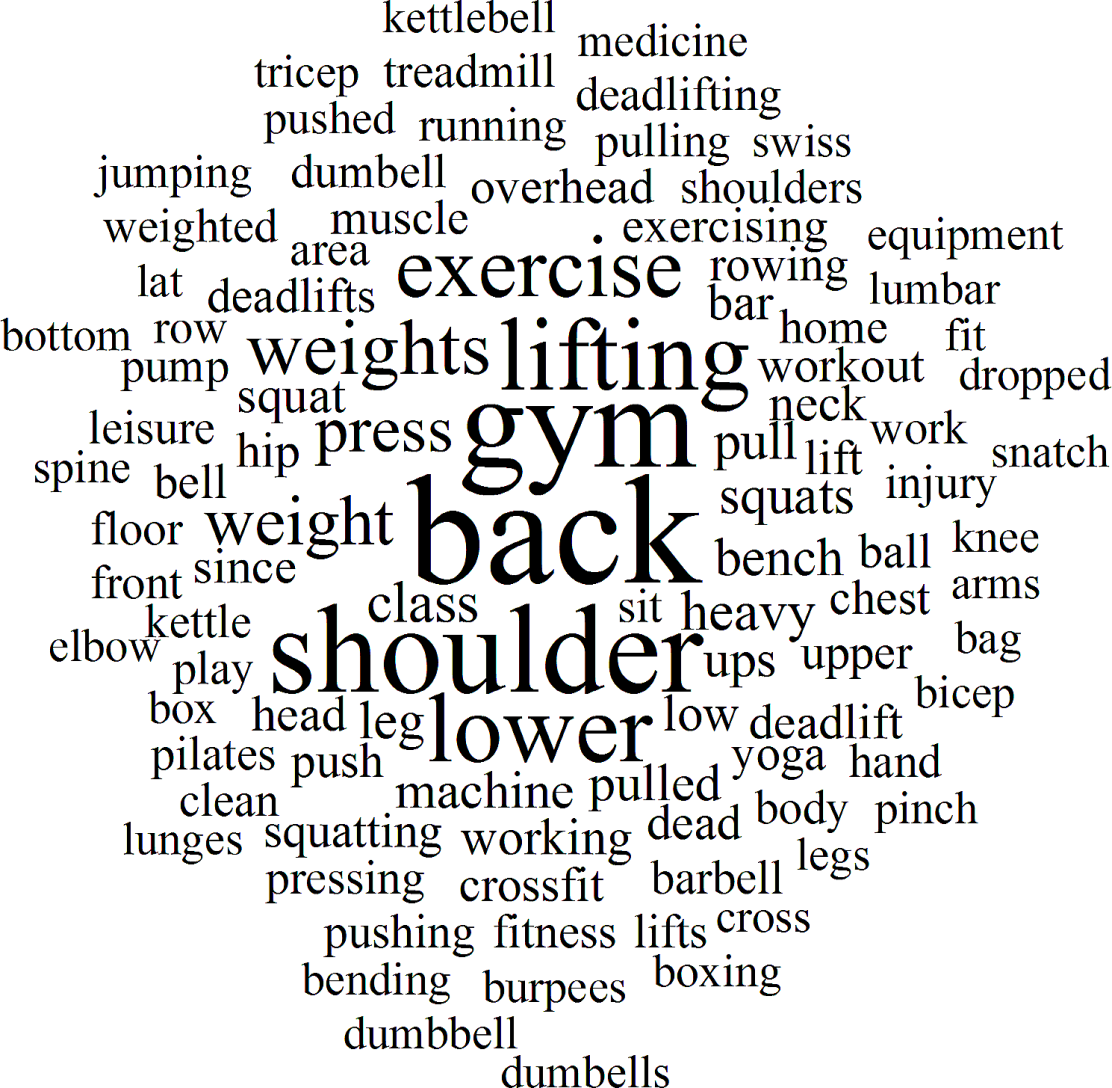
Word cloud of most frequent words in free text accident description

The 20 most frequent words extracted from the NVivo analyses are summarised in Table 6 and presented in a word cloud (Fig. 3). The most frequently recorded words were “back*” (*n* = 102,721, 11wt%) “gym*” (*n* = 82,250, 9wt%) “shoulder*” (*n* = 76,860, 8wt%) and “lift*” (*n* = 59,394, 6wt%). The 20 most frequent words were also tabulated into body part/location, movement/exercise type and activity and summarised in Table 7. The top exercises described were squat*, bench* press*, deadlift*. Shoulder*, lower back and leg* were the most common body parts described. It should also be noted that shoulder* could also be combined with press* to describe a shoulder press being performed at the time of injury. Gym*, lift* and weight* were the most common activities described in the free text entries.

**Table 6:** Word frequency of free text “accident description” field

Word	Count	Weighted percent (wt%)
Back*	102,721	11
Gym*	82,250	9
Shoulder*	76,860	8
Lift*	59,394	6
Low*	56,179	6
Weight*	54,967	6
Exercise*	43,757	5
Press*	22,986	2
Squat*	18,151	2
Pull*	17,031	2
Class*	11,844	1
Heavy*	10,708	1
Deadlift*	10,074	1
Leg*	8,776	1
Bench*	8,581	1
Work*	7,632	< 1
Machine*	7,038	< 1
Push*	6,966	< 1
Up*	6,511	< 1
Bar*	5,809	< 1

* denotes stemming

**Table 7:** Word frequency categorised

Body Part/Location	Movement/Exercise	Activity
Back*	Press*	Gym*
Shoulder*	Squat*	Lift*
Low*	Bench*	Weight*
Leg*	Pull*	Exercise*
	Deadlift*	Class*
	Up*	Heavy*
	Push*	Work*
		Machine*
		Bar*

* denotes stemming

## Discussion

This epidemiological study aimed to analyse gym and fitness related injury claims in New Zealand reported to ACC between 1 July 2011 and 30 June 2020 by reporting the prevalence, types, and causes of injury.

### Prevalence and Types of Gym Injuries

Analysis revealed a noteworthy presence (96%) of soft tissue injury claims with 48% caused by lifting/carrying/strain, and 71% of contact injury claims being caused by strenuous movements. The prevalence of injury claims reported to ACC caused by lifting and strenuous movement, aligned with findings from previous studies conducted in the Netherlands [32] and Australia [33] that highlighted strength training injuries as the most common gym injury presented. These findings emphasise the importance of addressing weightlifting related injuries.

Analysis of injury location demonstrated a wide range of body parts affected. The most frequently injured body sites of lower back, shoulder, knee and neck/back of head confirmed findings from previous studies [34]. The most common cause of soft tissue injury reported to ACC was lifting/carrying/strain (49%) and the most common contact reported was strenuous movement with lifting (47%). These findings align with hospital admissions in Victoria, Australia over an eight year period [35] and a recent study conducted in the Netherlands which stated strength training was the highest activity that resulted in injury [32]. Addressing injuries sustained to the lower back, shoulder, knee and neck/back of head accounts for approximately 65% of injuries and 76% of claim costs reported in New Zealand.

### Risk and Contributing Factors

Understanding the risk factors associated with gym related injuries could provide valuable insights to develop injury prevention initiatives. Survey information from the Australian quarterly exercise, recreation and sport survey has been extrapolated to calculate participation-adjusted injury incidence rates [33]. However, participation information was not available in New Zealand therefore incidence rates were not calculated for the New Zealand study. Moving forward it would be mutually beneficial for fitness facilities and ACC to collaborate more closely to establish gym participation data to inform future injury prevention research. Contributing factors to gym injuries have been discussed within the literature with causes of injury often driven by overuse, high loads, insufficient recovery, improper technique, or lack of expert supervision [32–34]. This highlights the importance of accessibility to exercise guidelines, safety considerations and coaching of proper form and technique especially in new and inexperienced gym users. Education and awareness of the value of varying the types of exercise performed (cross training) is important for injury prevention [36].

### Injury Prevention and Management

The findings of this study highlight the epidemiology of gym and fitness related injuries. Education of risks and emphasis on proper technique and form are critical and need to be accessible for all gym users. Furthermore, ensuring that gym staff and trainers are properly trained on observation and injury prevention. In addition, gathering as much quality information as possible when injuries occur, both within fitness facilities and by reporting agencies such as ACC, can provide valuable data for ongoing injury monitoring and formulation of trends.

### Data Limitations and Future Direction

We acknowledge the data limitations. Firstly, soft tissue injuries were not subcategorised, therefore may not have captured the full extent of certain types and severity of injuries. Additionally, the data did not capture exposure nor participation data. The ACC database recorded the number of injury claims but does not collect details of missed training time, hospitalization duration or level of participation. As in our prior studies [12, 37–39] we note that epidemiological studies are dependent on the data quality for analyses to be undertaken [40]. The ACC database was utilized as there were no other available databases for collection of fitness and gym specific data such as numbers participating in the different gym activities, age of gym goers participating, identification of the ethnicity of gym goers, and number of training sessions completed enabling calculation of training exposure hours. For confidentiality reasons, any data less than, or equal to, three injury claims were rounded to represent three claims to prevent reidentification of data. Costs and charges associated with each injury are not final and any future entitlements would result in the reported data herein to change. Finally, the ACC database only recorded the activity prior to the accident as “recreation/sporting activity”, and not if the claimant was exercising at the time of injury.

Future research could add more categories to the ACC claim form for sports injury to enable recording of participation data for calculation of risk factor. Our findings of free text entry analysis for word frequency highlighted key words that enabled an understanding of the activity being performed at the time of injury, however free text entries are not compulsory and were not completed for all data points. Whilst we reported some claim costs for the dataset, the aim was not to draw conclusions from the costs, therefore costs were not normalised for inflation. Further analysis, and breakdown of costs per year might be useful to make comparisons and determine trends over the given time period. Further important research questions regarding the mechanism of injury could be answered by performing a more in-depth thematic analysis [41, 42]. A prevention program aimed at addressing injuries of the lower back, shoulder, knee and neck/back of head could reduce the burden to ACC by focussing on 65% of the most frequently injures body parts which account for approximately 76% of claim costs.

## Conclusions

The most common cause of injury from gym and fitness activity claims in 16–64 year olds in New Zealand was lifting/carrying/strain resulting in lower back/spine and shoulder (including clavicle/blade) soft tissue injuries. Soft tissue injuries accounted for 96% of the total claims. Males and females aged 21 to 30 years old were most frequently injured. Exposure and participation data are needed to calculate injury risk of gym and fitness activities, which highlights opportunity for collaboration between gyms and ACC.

### Electronic supplementary material

Below is the link to the electronic supplementary material.

Supplementary material 1

## Data Availability

Due to restrictions on use of data stated in the data sharing agreement, the research team are not able to share the data used for these analyses. However, data sets generated and analysed during the current study may be requested via ACC.

## Abbreviations

ACC: Accident Compensation Corporation
MELAA: Middle Eastern/Latin American/African
MSC: Moderate-to-serious claims
n: Number
NZ: New Zealand
NZ$: New Zealand Dollar currency
REP’s: Register of Exercise Professionals
