# Supplementary material for: Gym and Fitness Injuries amongst those Aged 16–64 in New Zealand: Analysis of Ten Years of Accident Compensation Corporation Injury Claim Data
Source: Sports Med Open. 2024 May 14;10:53. doi: 10.1186/s40798-024-00694-9 (PMC11093940; doi:10.1186/s40798-024-00694-9)
Supplement: Supplementary file 1 — Supplementary material 1 [file 40798_2024_694_MOESM1_ESM.docx]

**Supplementary Tables**

**Supplementary Table 1.** Data categories obtained from ACC.

| **Ethnicity** |
| --- |
| New Zealand European |
| New Zealand Māori |
| Pacific Peoples |
| Cook Island Māori |
| Samoan  Tongan |
| Niuean |
| Fijian |
| Tokelauan |
| Other Pacific |
| Asian |
| South-East Asian |
| Indian |
| Other/Unknown |
| **Region** |
| North Island |
| Northland |
| Auckland |
| Waikato |
| Bay of Plenty |
| Gisborne |
| Hawke’s Bay |
| Taranaki |
| Manawatu-Wanganui Wellington |
| South Island |
| Tasman |
| Nelson |
| Marlborough |
| Canterbury |
| West Coast |
| Otago |
| Southland |
| Other/Unknown |
| **Injury site** |
| Head/Neck |
| Head (except face) |
| Face |
| Eye |
| Nose |
| Ear |
| Neck / back of head / vertebrae |
| Upper Limb |
| Shoulder (including clavicle/shoulder blade) upper and lower arm |
| Elbow |
| Hand / wrist |
| Finger / thumb |
| Lower Limb |
| Hip / upper leg / thigh knee |
| Lower Limb |
| Ankle |
| Foot |
| Toes |
| Other |
| Abdomen / pelvis |
| Chest |
| Lower back / spine |
| Lung / internal organ |
| Multiple locations |
| Unknown |
| **Injury type** |
| Soft tissue injury |
| Burns |
| Compression syndrome |
| Contusion |
| Fracture / dislocation |
| Laceration, puncture, wound, sting |
| Localised inflammation |
| Pain syndrome |
| Trauma induced hearing loss |
| Other / unknown |
| **Side of body** |
| Right |
| Left |
| Not applicable |
| **Cause** |
| Boiling (violent & inadvertent) |
| Bursting / breakage / distortion |
| Collapse of stack / bulk goods |
| Collapse / overturning |
| Collision / knocked over by object |
| Driving into hole / object |
| Electrical shock / short circuit |
| Explosion / blasting / implosion |
| Exposure to elements |
| Fire |
| Flooding / overflow of liquid |
| Folding / collapse |
| Inadvertent machine / vehicle movement |
| Lifting / carrying / strain |
| Loss balance / personal control |
| Loss of consciousness / Sleep |
| Loss of control of vehicle |
| Loss of hold |
| Lurching / Jerks in vehicles etc |
| Mechanical malfunction |
| Medical treatment |
| Misjudgement of support |
| Object coming loose / shifting |
| Oral ingestion of fungi |
| Other or unclear cause |
| Puncture |
| Pushed or pulled |
| Recoil / ejection |
| Shooting |
| Skid |
| Slipping, Skidding on Foot |
| Something Giving way Underfoot |
| Struck by held tool / Implement |
| Struck by person / Animal |
| Swerving / evasive action |
| Tripping or stumbling |
| Twisting movement |
| Unclear fire or explosion |
| Weak property or characteristics |
| **Contact** |
| Strenuous movement without lifting |
| Impact / contact with object |
| Impact / contact with ground / Floor |
| Other or unclear contact |
| Repetitive movement |
| Caused own injury without tool |
| Contact while handling / carrying |
| Other contact with person |
| Dropped object carried / handled |
| Contact with object carried / handled |
| Step on sharp object |
| Falling objects not handled |
| Other moving object / part etc |
| Contact with moving object |
| Collision |
| Turning over / capsizing |
| Liquid, Powder at Rest |
| Drove into object / ditch / falling |
| Hit Vehicle / Ground while in vehicle |
| Kicked / Butted / Bitten by animal |
| Medical treatment |
| Inhaled / swallowed substance / object |
| Exposed to Flame / Noise / Elect |
| Environmental elements |
| Fight / Struggle with person |
| Witnessed |
| Flying Object / Spatter / Fragments |

**Supplementary Table 2**. Descriptions of soft tissue injury within relevant injury literature.

| Schwellnus, 2011 [24] | Muscular, tendinous, ligamentous, vascular, and nervous tissue |
| --- | --- |
| Ball et. al., 2017 [25] | Joint (non-bone) and ligament (dislocation, subluxation, sprain, ligament injury, lesion of meniscus/cartilage/disc); muscle and tendon (muscle rupture/tear/strain/cramp, tendon injury/ rupture/tendinopathy/bursitis, haematoma, contusion, bruise); skin (abrasion, laceration); and central nervous system (brain/ spinal cord)/peripheral nervous system (CNS/PNS) (concussion, structural brain injury, spinal cord compression/transection, nerve injury) |
| Nyland et. al., 2000 [45] | Strain, sprain, tendonitis, bursitis, or contusion |
| Best, 1997 [46] | Acute strain injury, contusion, delayed onset muscle soreness (DOMS), acute compartment syndrome, chronic compartment syndrome, muscle cramps, rhabdomyolysis. |

**Supplementary Table 3.** Injury claims and population distribution by region.

| **Region** | **Number of injury claims** | **% Injuries** | **Population** | **% Population** |
| --- | --- | --- | --- | --- |
| Auckland | 15,6035 | 45 | 1,571,718 | 33 |
| Canterbury | 36,106 | 10 | 599,694 | 13 |
| Wellington | 40,578 | 12 | 506,814 | 11 |
| Waikato | 26,284 | 8 | 458,202 | 10 |
| Bay of Plenty | 19,234 | 6 | 308,499 | 7 |
| Manawatu-Wanganui | 9,149 | 3 | 238,797 | 5 |
| Otago | 19,299 | 6 | 225,186 | 5 |
| Northland | 7,655 | 2 | 179,076 | 4 |
| Hawkes Bay | 10,825 | 3 | 166,368 | 3.5 |
| Taranaki | 5,087 | 1 | 117,561 | 2.5 |
| Southland | 4,057 | 1 | 97,467 | 2 |
| Tasman | 1,237 | <1 | 52,389 | 1 |
| Nelson | 4,109 | 1 | 50,880 | 1 |
| Gisborne | 2,454 | <1 | 47,517 | 1 |
| Marlborough | 1,768 | <1 | 47,340 | 1 |
| West Coast | 637 | <1 | 31,575 | <1 |
| Other | 740 | <1 | 669 | <1 |

**Supplementary Table 4**. Soft tissue injury claims (n=331,343) by body site, cost, and frequency of claims.

| **Body site** | **Cost (NZ$)** | **Frequency** |
| --- | --- | --- |
| Lower back/spine | 57,111,225 | 81,799 |
| Shoulder (incl clavicle/blade) | 52,245,523 | 61,784 |
| Knee | 31,010,754 | 36,153 |
| Neck, back of head vertebrae | 19,125,569 | 37,845 |
| Hip, upper leg, thigh | 11,479,355 | 23,651 |
| Ankle | 9,993,530 | 16,363 |
| Hand/wrist | 6,952,483 | 10,174 |
| Upper and lower arm | 6,024,841 | 11,414 |
| Upper back/spine | 5,329,567 | 17,610 |
| Lower leg | 3,287,925 | 10,228 |
| Abdomen/pelvis | 2,629,158 | 5,455 |
| Foot | 2,524,913 | 6,158 |
| Finger/thumb | 1,402,267 | 3,016 |
| Elbow | 1,258,815 | 2,631 |
| Chest | 1,132,226 | 4,196 |
| Unobtainable | 762,158 | 1,427 |
| Toes | 243,241 | 741 |
| Head (except face) | 218,918 | 134 |
| Face | 116,828 | 277 |
| Multiple locations | 112,515 | 107 |
| Nose | 51,417 | 87 |
| Eye | 23,122 | 69 |
| Other internal organ | 10,434 | 3 |
| Ear | 1,986 | 15 |
| Internal organ | 428 | 6 |
| Total | 213,049,197 | 331,343 |

**Supplementary Table 5.** Cost and frequency by sex of top 10 soft tissue (n=331,343) injury sites. * Note the percentages (%) do not add up to 100% as the table represents only the top ten injury sites reported in the data set.

| **Injury site** | **Total cost NZ$** | **Total number** | **Female n** | **Female %*** | **Male n** | **Male %*** |
| --- | --- | --- | --- | --- | --- | --- |
| Lower back/spine | 57,506,387 | 81,846 | 39,589 | 23 | 42,257 | 25 |
| Shoulder/clavicle | 56,197,315 | 62,760 | 26,535 | 15 | 36,225 | 21 |
| Knee | 32,018,328 | 37,035 | 22,108 | 13 | 14,927 | 9 |
| Neck/ back of head/vertebrae | 19,247,382 | 37,860 | 20,847 | 12 | 17,013 | 10 |
| Hip/thigh/upper leg | 12,883,922 | 23,871 | 13,774 | 8 | 10,097 | 6 |
| Unobtainable | 10,971,005 | 3,599 | 16,25 | 1 | 1,974 | 1 |
| Ankle | 10,735,480 | 16,594 | 10,623 | 6 | 5,970 | 4 |
| Hand/wrist | 8,735,453 | 10,895 | 5,228 | 3 | 5,667 | 3 |
| Upper/lower arm | 7,584,137 | 12,243 | 4,415 | 3 | 7,828 | 5 |
| Upper back/spine | 5,384,910 | 17,622 | 8,457 | 5 | 9,165 | 5 |
